# Serum Concentrations of Ischaemia-Modified Albumin in Acute Coronary Syndrome: A Systematic Review and Meta-Analysis

**DOI:** 10.3390/jcm11144205

**Published:** 2022-07-20

**Authors:** Arduino A. Mangoni, Angelo Zinellu

**Affiliations:** 1 Discipline of Clinical Pharmacology, College of Medicine and Public Health, Flinders University, Bedford Park, SA 5042, Australia; 2 Department of Clinical Pharmacology, Flinders Medical Centre, Southern Adelaide Local Health Network, Flinders Drive, Bedford Park, SA 5042, Australia; 3 Department of Biomedical Sciences, University of Sassari, 07100 Sassari, Italy; azinellu@uniss.it

**Keywords:** ischaemia-modified albumin, acute coronary syndrome, acute myocardial infarction, unstable angina, non-ST-elevation myocardial infarction, ST-elevation myocardial infarction, biomarkers

## Abstract

The identification of novel circulating biomarkers of acute coronary syndrome (ACS) may improve diagnosis and management. We conducted a systematic review and meta-analysis of ischaemia-modified albumin (IMA), an emerging biomarker of ischaemia and oxidative stress, in ACS. We searched PubMed, Web of Science, and Scopus from inception to March 2022, and assessed the risk of bias and certainty of evidence with the Joanna Briggs Institute Critical Appraisal Checklist and GRADE, respectively. In 18 studies (1654 ACS patients and 1023 healthy controls), IMA concentrations were significantly higher in ACS (standard mean difference, SMD = 2.38, 95% CI 1.88 to 2.88; *p* < 0.001; low certainty of evidence). The effect size was not associated with pre-defined study or patient characteristics, barring the country where the study was conducted. There were no significant differences in effect size between acute myocardial infarction (MI) and unstable angina (UA), and between ST-elevation (STEMI) and non-ST-elevation MI (NSTEMI). However, the effect size was progressively larger in UA (SMD = 1.63), NSTEMI (SMD = 1.91), and STEMI (3.26). Our meta-analysis suggests that IMA might be useful to diagnose ACS. Further studies are warranted to compare the diagnostic performance of IMA vs. established markers, e.g., troponin, and to determine its potential utility in discriminating between UA, NSTEMI, and STEMI (PROSPERO registration number: CRD42021324603).

## 1. Introduction

Acute coronary syndrome (ACS) and its traditional subtypes, unstable angina (UA), non-ST-elevation myocardial infarction (NSTEMI), and ST-elevation myocardial infarction (STEMI), remain a leading cause of morbidity and mortality worldwide [1,2]. The early diagnosis of UA, NSTEMI, and STEMI using clinical assessment, biomarkers of myocardial injury and imaging studies is essential for appropriate management and favourable outcomes [3,4]. Available circulating biomarkers of ACS, e.g., cardiac troponin, have transformed ACS management, prognostication, and resource allocation [5]. However, there is an ongoing search for additional biomarkers to provide valuable mechanistic insights, and further improve diagnostic and prognostic accuracy [6,7]. Ideally, such biomarkers should be rapidly measurable and easily interpretable using robust and reproducible analytical methods [8].

Several proteins have been investigated as potential ACS biomarkers in view of their ability to reflect critical pathophysiological processes, e.g., atherosclerotic plaque instability, inflammation, myocardial cell injury, haemodynamic stress, and altered metabolism [6,7]. One such protein, albumin, has been shown to undergo chemical modifications, albeit the exact reactions remain elusive, during ischaemic states. These changes, likely the consequence of a state of acidosis and oxidative stress, combined with the production of reactive oxygen species, lead to the generation of ischaemia-modified albumin (IMA) [9]. Notably, IMA is transient, and generally reverts to albumin within 24 h after ischaemia in studies of balloon occlusion during percutaneous coronary intervention [10]. Furthermore, there is increased generation of IMA with relatively longer periods of ischemia [9].

Serum concentrations of IMA have been detected three hours after the onset of ACS symptoms, with a sensitivity of 70%, a specificity of 80%, and a positive predictive value of 96%, suggesting the potential role of this protein as a biomarker of ACS [11]. Therefore, we critically appraised the available evidence regarding the association between IMA and ACS by conducting a systematic review and meta-analysis of serum IMA concentrations in ACS patients and healthy controls. The primary hypothesis was that IMA concentrations were significantly higher in ACS. In addition, we sought to determine the presence of differences in IMA concentrations between the main ACS subtypes: UA, NSTEMI, and STEMI.

## 2. Materials and Methods

### 2.1. Literature Search and Study Selection

We searched articles in PubMed, Web of Science, and Scopus, from inception to March 2022, using the following terms: “acute coronary syndrome” or “ACS” or “acute myocardial infarction” or “AMI” or “unstable angina” or “UA” or “non-ST-elevation myocardial infarction” or “NSTEMI” or “ST-elevation myocardial infarction” or “STEMI” and “ischaemia modified albumin” or “IMA”. The abstracts were independently screened by two investigators and, if relevant, the full text was reviewed. Eligibility criteria were: (i) assessment of IMA; (ii) comparison of subjects with or without ACS or its sub-types (case–control design); (iii) use of English language; (iv) availability of the full text. The references of the retrieved articles were also searched to identify additional studies. Any between-reviewer disagreement was resolved by a third investigator. The following information was extracted from each article: age, proportion of males, year of publication, country where the study was conducted, sample size, IMA concentrations, serum troponin concentrations, and ACS subtype. The Joanna Briggs Institute (JBI) Critical Appraisal Checklist for analytical studies was used to assess the risk of bias (a low, moderate, and high risk of bias was indicated by a score of ≥5, 4, and <4, respectively) [12]. The Grades of Recommendation, Assessment, Development and Evaluation (GRADE) was used to assess the certainty of evidence. GRADE considers the risk of bias, presence of unexplained heterogeneity, indirectness of evidence, imprecision of the results, effect size [13], and probability of publication bias [14]. The study adhered to the Preferred Reporting Items for Systematic reviews and Meta-Analyses (PRISMA) 2020 statement (Supplementary Tables S1 and S2) [15]. The protocol was registered in the International Prospective Register of Systematic Reviews (PROSPERO, CRD42021324603).

### 2.2. Statistical Analysis

Standardised mean differences (SMDs) and 95% confidence intervals (CIs) were used to build forest plots of continuous data, and to evaluate differences in IMA concentrations between participants with or without ACS (significance level at *p* < 0.05) as different units of measurement (U/mL, absorbance units, pg/mL, or g/dL) were used. SMD heterogeneity was tested using the Q statistic (significance level at *p* < 0.10). An I^2^ value < 30% indicated no or slight heterogeneity, whereas I^2^ ≥ 30% indicated moderate or substantial heterogeneity [16]. A random-effect model was used in presence of moderate or substantial heterogeneity [16]. Sensitivity analysis investigated the influence of each study on the overall risk estimate [17]. Begg’s and Egger’s tests were used to assess publication bias (significance level at *p* < 0.05) [18,19]. The Duval and Tweedie “trim-and-fill” procedure was used to attempt to correct the publication bias [20]. Subgroup analyses were conducted to investigate possible differences in effect size according to ACS subtypes and country where the study was conducted. Associations between effect size, and study and patient characteristics (age, proportion of males, year of publication, country where the study was conducted, sample size, and serum troponin concentrations) were also investigated using univariate meta-regression analysis. Statistical analyses were performed using Stata 14 (Stata Corp., College Station, TX, USA).

## 3. Results

### 3.1. Systematic Research

**Figure 1:**
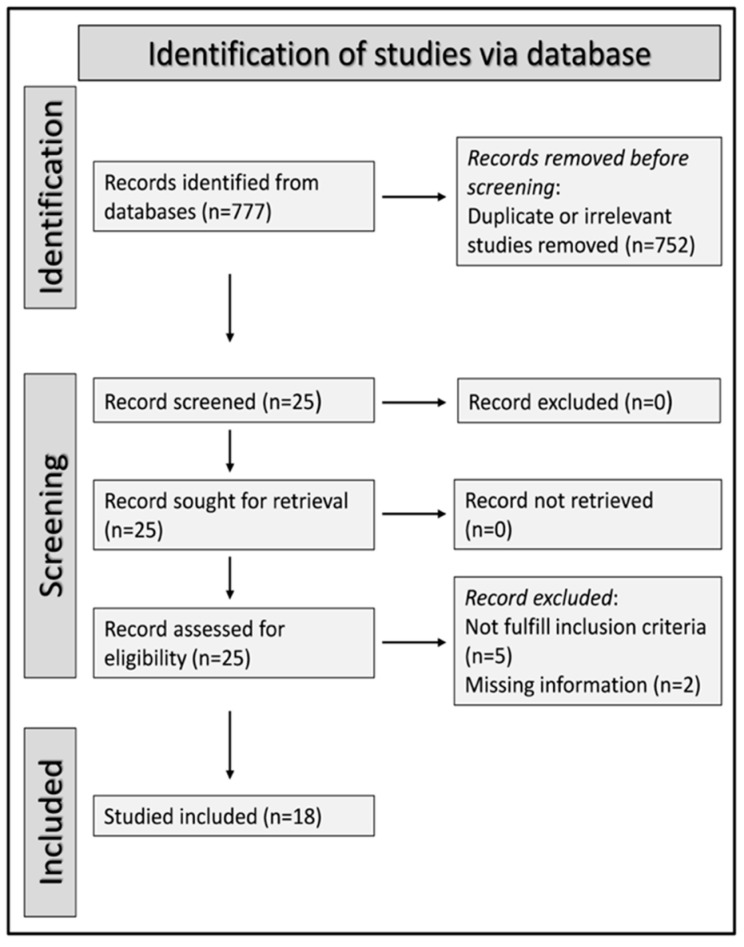
PRISMA 2020 flow diagram.

A PRISMA 2020 flow chart is presented in Figure 1. After initially identifying 777 studies, 752 were excluded (either duplicates or irrelevant). After a full-text review of the remaining 25 articles, seven were further excluded because they did not fulfil the inclusion criteria or presented duplicate data, leaving 18 studies for analysis (Figure 1).

### 3.2. Studies Selected

The 18 selected studies included 32 comparator arms in 1654 ACS patients (mean age 60 years, 60% men) and 1023 healthy controls or subjects with atypical chest pain without ACS (mean age 53 years, 58% men) (Table 1) [11,21,22,23,24,25,26,27,28,29,30,31,32,33,34,35,36,37]. IMA was measured using automated analysers in two studies [24,35], enzyme-linked immunosorbent assay in one [31], and the spectrophotometric albumin cobalt binding assay in the remaining 15 [11,21,22,23,25,26,27,28,29,30,32,33,34,36,37]. Six comparative arms investigated overall ACS [21,22,25,28,29,34], two overall AMI [24,26], seven STEMI [11,27,30,31,33,35,36], nine NSTEMI [11,27,30,31,32,33,35,36,37], and eight UA [11,23,26,27,30,31,33,35]. In all studies, IMA was measured within 24 h of the onset of symptoms [11,21,22,23,24,25,26,27,28,29,30,31,32,33,34,35,36,37].

### 3.3. Risk of Bias

The risk of bias was low in all studies (Supplementary Table S3).

### 3.4. Results of Individual Studies and Syntheses

**Figure 2:**
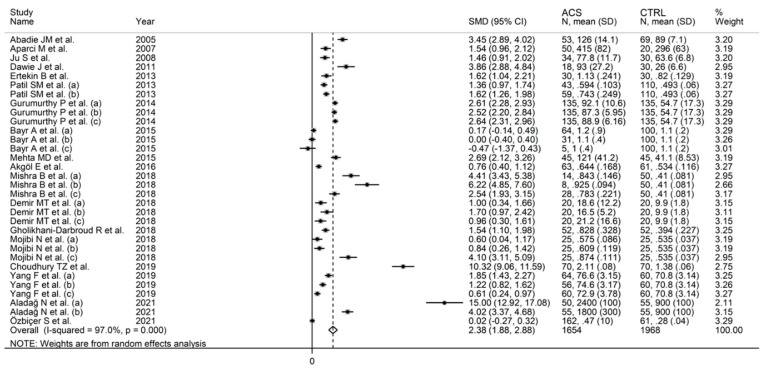
Forest plot of studies examining ischaemia-modified albumin in patients with acute coronary syndrome and controls.

The forest plot of IMA concentrations in ACS patients and controls is shown in Figure 2. In two comparator arms from the same study, IMA concentrations were either lower in ACS patients, or virtually identical between ACS patients and controls [27]. In the remaining comparator arms, ACS patients had higher IMA concentrations, although the difference was not significant in two [27,37]. There was substantial heterogeneity (I^2^ = 97%, *p* < 0.001). Pooled results showed that IMA concentrations were significantly higher in ACS (SMD = 2.38, 95% CI 1.88 to 2.88; *p* < 0.001).

**Figure 3:**
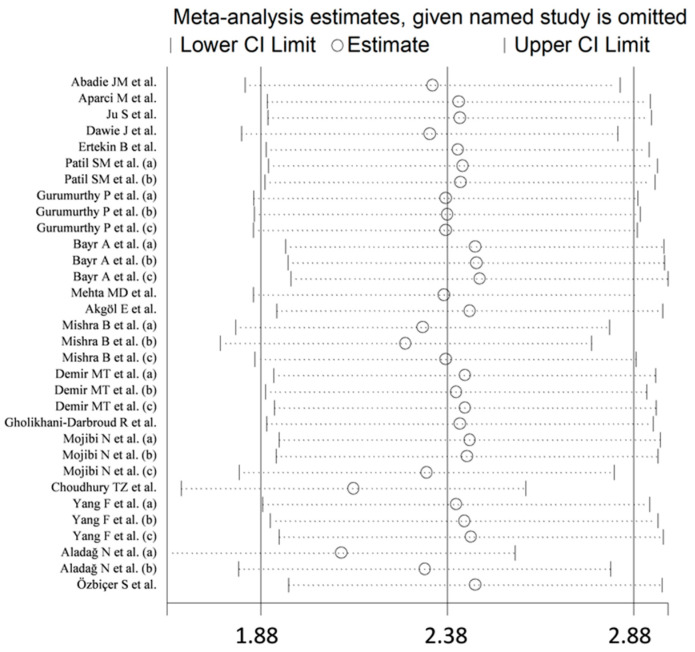
Sensitivity analysis of the association between ischaemia-modified albumin and acute coronary syndrome. For each study, the effect size (hollow circles) corresponds to an overall effect derived from a meta-analysis excluding that study.

**Figure 4:**
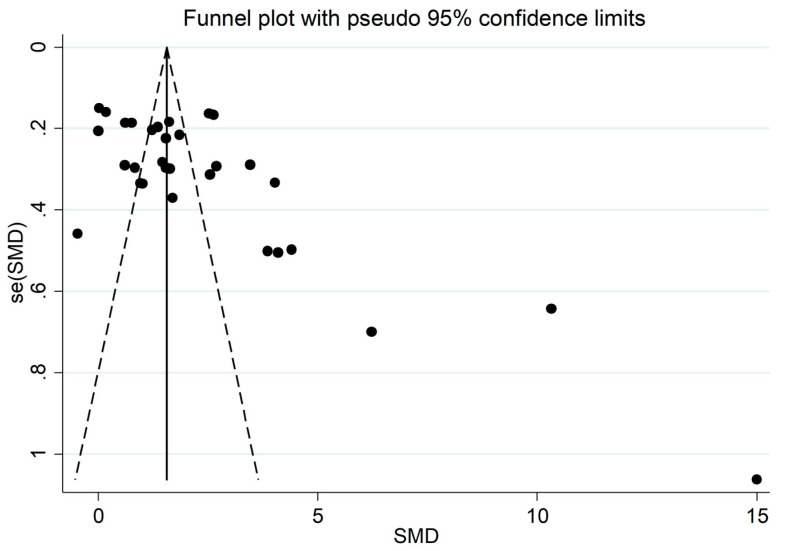
Funnel plot of studies investigating ischaemia-modified albumin concentrations in patients with acute coronary syndrome and controls.

In the sensitivity analysis, the corresponding pooled SMDs were not substantially altered when individual studies were omitted (effect size range, between 2.10 and 2.47, Figure 3). The funnel plot in Figure 4 revealed a distortive effect of three studies [30,34,36]. Their removal attenuated the effect size (SMD-1.74, 95% CI 1.32 to 2.15; *p* < 0.001) but not the heterogeneity (I^2^ = 95.7%, *p* < 0.001).

### 3.5. Publication Bias

**Figure 5:**
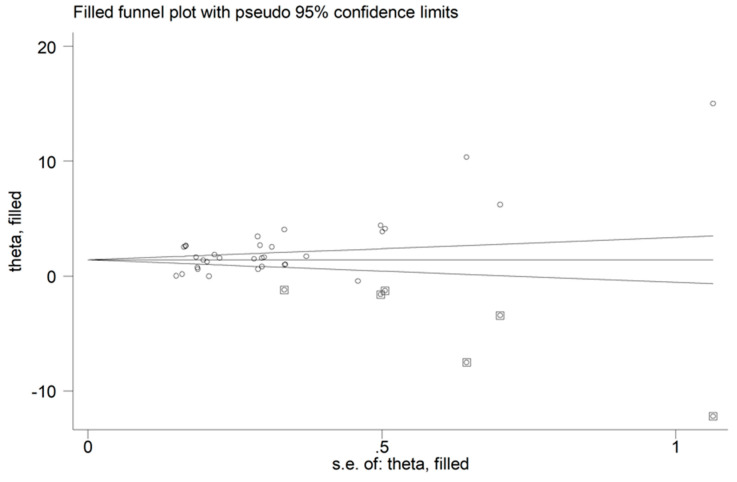
Funnel plot of ischaemia-modified albumin concentrations in patients with acute coronary syndrome and controls after “trimming-and-filling”. Dummy studies and genuine studies are represented by enclosed circles and free circles, respectively.

There was a significant publication bias found, according to Begg’s (*p* = 0.009) and Egger’s tests (*p* = 0.001). The “trim-and-fill” method identified six potential missing studies to be added to the left of the funnel plot to ensure symmetry (Figure 5). This resulted in a reduced, albeit significant, effect size (SMD = 1.46, 95% CI 0.91 to 2.01; *p* < 0.001).

### 3.6. Subgroup Analysis and Meta-Regression

**Figure 6:**
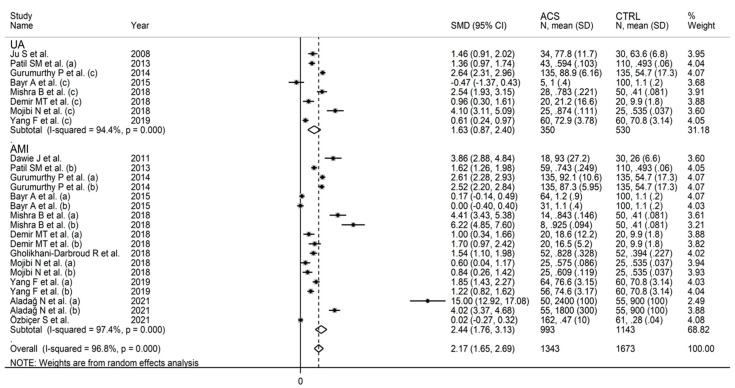
Forest plot of studies examining ischaemia-modified albumin in patients with acute myocardial infarction or unstable angina vs. controls.

**Figure 7:**
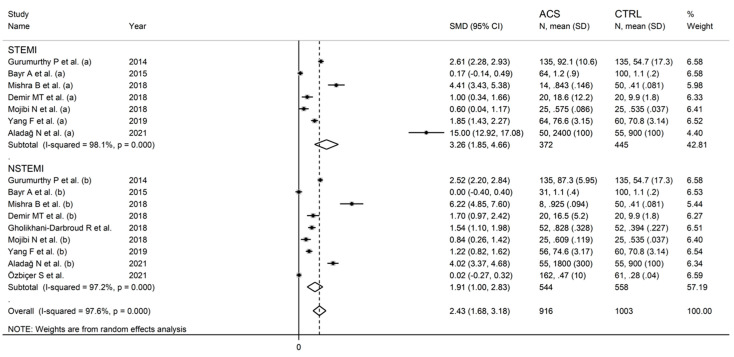
Forest plot of studies examining ischaemia-modified albumin in ST-elevation or non-ST-elevation myocardial infarction vs. controls.

**Figure 8:**
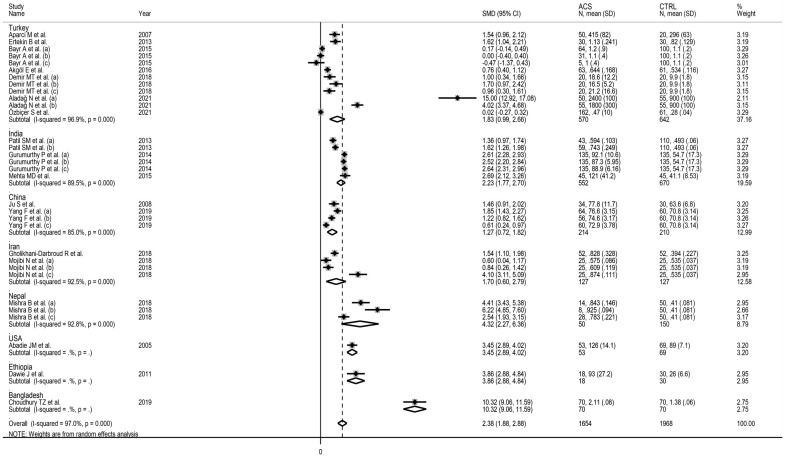
Forest plot of studies examining ischaemia-modified albumin according to the country where the study was conducted.

As reported in Figure 6, IMA was able to discriminate between UA patients and healthy controls, and between AMI patients and healthy controls. The effect size was relatively, albeit non-significantly (*p* = 0.43), larger in AMI (SMD = 2.44, 95% CI 1.76 to 3.13; *p* < 0.001) than UA (SMD = 1.63, 95% CI 0.87 to 2.40; *p* < 0.001). Heterogeneity was substantial in both groups (94.4% and 96.8%). Similarly, IMA was able to discriminate between STEMI patients and healthy controls, and between NSTEMI patients and healthy controls (Figure 7). There were no significant differences (*p* = 0.42) in effect size between STEMI (SMD = 3.26, 95% CI 1.85 to 4.66; *p* < 0.001) and NSTEMI (SMD = 1.91, 95% CI 1.00 to 2.53; *p* < 0.001), with substantial study variance in both groups (98.1% and 97.2%). Albeit not significantly, the effect size was progressively larger in UA (SMD = 1.63), NSTEMI (SMD = 1.91), and STEMI (SMD = 3.26). The effect size was also relatively larger in studies conducted in Nepal (SMD = 4.32, 95% CI 2.27 to 6.36; *p* < 0.001) and India (SMD = 2.23, 95% CI 1.77 to 2.70; *p* < 0.001) when compared to Turkey (SMD = 1.83, 95% CI 0.89 to 2.66; *p* < 0.001), Iran (SMD = 1.70, 95% CI 0.60 to 2.79; *p* < 0.001) or China (SMD = 1.27, 95% CI 0.72 to 1.82; *p* < 0.001). Heterogeneity remained substantial, between 85.0% and 96.9%, in all sub-groups (Figure 8).

In univariate meta-regression, there were no significant associations between the effect size and age (*t* = −0.55, *p* = 0.59), proportion of males (*t* = 0.45, *p* = 0.66), publication year (*p* = 1.15, *p* = 0.26), sample size (*p* = −0.11, *p* = 0.91), or troponin concentrations (*p* = 1.45, *p* = 0.16). By contrast, a significant association was observed between the effect size and the country where the study was conducted (*p* = 2.19, *p* = 0.037).

### 3.7. Certainty of Evidence

The initial level of certainty for IMA SMD values was considered low because of the cross-sectional nature of the studies (rating 2, ⊕⊕⊝⊝). After taking into account the low risk of bias in all studies (no rating change), the substantial and unexplained heterogeneity (downgrade one level), the lack of indirectness (no rating change required), the relatively low imprecision (relatively narrow confidence intervals without threshold crossing, no rating change required), the large effect size (SMD = 2.38, upgrade one level), and the presence of publication bias which was addressed with the “trim-and-fill” method (no rating change), the overall level of certainty remained low (rating 2, ⊕⊕⊝⊝).

## 4. Discussion

Our systematic review and meta-analysis have shown that serum IMA concentrations are significantly higher in patients with ACS, when measured within 24 h of the onset of symptoms, compared to healthy controls or subjects with atypical chest pain without ACS. In subgroup analysis, there were no significant differences in effect size between AMI vs. controls and UA vs. controls, and between STEMI vs. controls and NSTEMI vs. controls. The effect size was progressively, albeit not significantly, larger in UA vs. NSTEMI vs. STEMI, the three traditional subtypes of ACS. Furthermore, the effect size was relatively larger in studies conducted in Nepal and India when compared to those conducted in Turkey, Iran, or China. Barring the country where the study was conducted, in meta-regression the effect size was not significantly associated with a number of study and patient characteristics, including serum troponin concentrations. Therefore, the results of our study suggest that IMA could be a useful biomarker for the early diagnosis of ACS and complement those of a recent meta-analysis investigating the diagnostic accuracy of IMA in ACS. This meta-analysis reported a pooled odds ratio of 3.72, an area under the curve of 0.75, a sensitivity of 0.74, and a specificity of 0.40 [38].

Appropriately designed studies are also warranted to determine the potential utility of IMA in discriminating between UA, NSTEMI, and STEMI, although the routine use of high-sensitivity troponin will likely lead to the incorporation of UA into NSTEMI in the foreseeable future [3,4,5]. In this context, however, the lack of significant associations in meta-regression between IMA and troponin suggests that the diagnostic and pathophysiological information provided by IMA might complement, rather than duplicate, that provided by troponin in ACS. More research is needed to address this issue and determine the utility of routinely measuring IMA in this patient group.

Several colorimetric and immunochemical methods are available to measure IMA. Some of them, e.g., the albumin copper-binding assay, the enzyme-linked immunosorbent assay, and the surface plasmon resonance immunosensor, are relatively simple and have high sensitivity and specificity [9]. In our systematic review and meta-analysis, the albumin cobalt-binding method, based on the measurement of the binding of cobalt to albumin in serum, was used in 15 out of 18 studies [39]. However, this method has limitations as the results can be affected by conformational changes in albumin due to changes in pH or presence of denaturing agents, chemicals, or medications [9]. Furthermore, the results are expressed as absorbance units, which might be influenced by investigator experience and/or sensitivity of the equipment, and some investigators have used internal standards for IMA obtained in their laboratories [9]. These issues might account, at least partly, for the substantial study heterogeneity observed in our analyses. The relatively larger effect size observed in studies conducted in Nepal and India, compared to those conducted in Turkey, Iran, or China, highlights possible ethnic-related differences in IMA production, as has also been reported in other studies [40,41]. This issue warrants further research in prospective studies that include an ethnically diverse population.

It is important to emphasise that IMA can also be generated in non-ischaemic conditions that are characterised by various degrees of oxidative stress, e.g., heart failure [42], neurodegenerative diseases [43], diabetes [44], pregnancy disorders [45,46,47], and cancer [48]. Whilst the results of these studies suggest that IMA elevations are not specific to ACS, they also indicate that IMA generation might reflect the presence of a pro-oxidant state in the context of myocardial ischaemia, a well-described phenomenon in animal models and humans [49,50,51]. Therefore, further research is warranted to investigate the role of IMA as a combined biomarker of myocardial damage and oxidative stress in patients with ACS, and whether specific temporal patterns of IMA concentrations reflect differences in response to revascularization strategies. These issues notwithstanding, IMA concentrations have been shown to be associated with outcomes in ACS. In a study of 207 patients presenting to the Emergency Department with acute chest paint suggestive of ACS, IMA concentrations on admission independently predicted a 30-day composite endpoint of cardiac death, AMI, or recurrent angina (odds ratio, OR, 1.04, 95% CI 1.01 to 1.07; *p* = 0.01) as well as one-year mortality (hazard ratio, HR, 1.038; 95% CI 1.006 to 1.070; *p* = 0.018) [52]. A recent systematic review and meta-analysis that also included this study has reported similar findings, with IMA concentrations significantly associated with major adverse cardiovascular events (OR 1.85, 95% CI 1.05 to 3.29; *p* = 0.03) [53].

The strengths of our study include the conduct of subgroup analyses for AMI/UA vs. controls and NSTEMI/STEMI vs. controls, the investigation of possible associations between the effect size and several patient and study characteristics with meta-regression, and the assessment of the certainty of evidence using GRADE. One limitation is that, barring two studies [21,24], the articles identified involved studies that were primarily conducted in Asian populations, which limits the generalizability of our findings to other ethnic groups. Although another important limitation is the substantial between-study heterogeneity, in sensitivity analysis the effect size was not substantially affected when individual studies were in turn removed.

## 5. Conclusions

Our systematic review and meta-analysis have shown the presence of significant differences in serum IMA concentrations between patients with ACS and healthy controls, or patients with atypical chest pain without ACS. Additional research is warranted to investigate the relationships between IMA generation and the extent of myocardial injury, the effect of revascularization strategies, short- and long-term outcomes, and other specific patient characteristics, including ethnicity. Importantly, these studies should also include patients presenting with typical and atypical chest pain. The results of these studies will determine the potential utility of IMA, singly or in combination with established biomarkers, e.g., high-sensitivity troponin, in the routine diagnosis, risk stratification, and prognosis in patients with ACS.

## Figures and Tables

**Table 1:** Study characteristics.

	Controls				Acute Coronary Syndrome				
First Author and Year, Country	N	Age *	M/F	IMA Mean ± SD	N	Age *	M/F	IMA Mean ± SD	Sub-Type
Abadie JM et al. 2005, USA [21]	69 #	49	NR	89 ± 7.2 U/mL	53	64	NR	126 ± 14.1 U/mL	ACS
Aparci M et al. 2007, Turkey [22]	20 ^	65	12/8	296 ± 63 U/mL	50	67	38/12	415 ± 82 U/mL	ACS
Ju S et al. 2008, China [23]	30 #	63	16/14	63.6 ± 6.8 AU/mL	34	68	18/16	77.8 ± 11.7 AU/mL	UA
Dawie J et al. 2011, Ethiopia [24]	30 #	NR	NR	26 ± 6.6 ACBU	18	NR	NR	93 ± 27.2 ACBU	AMI
Ertekin B et al. 2013, Turkey [25]	30 #	52	14/16	0.820 ± 0.129 ABSU	30	57	12/18	1.134 ± 0.241 ABSU	ACS
Patil SM et al. India, 2013 (a) [26]	110 #	40	67/43	0.493 ± 0.060 ABSU	43	43	31/12	0.594 ± 0.103 ABSU	UA
Patil SM et al. India, 2013 (b) [26]	110 #	40	67/43	0.493 ± 0.060 ABSU	59	49	43/16	0.743 ± 0.249 ABSU	AMI
Gurumurthy P et al. India, 2014 (a) [11]	135 #	NR	NR	54.7 ± 17.29 U/mL	135	NR	NR	92.1 ± 10.6 U/mL	STEMI
Gurumurthy P et al. India, 2014 (b) [11]	135 #	NR	NR	54.7 ± 17.29 U/mL	135	NR	NR	87.31 ± 5.95 U/mL	NSTEMI
Gurumurthy P et al. India, 2014 (c) [11]	135 #	NR	NR	54.7 ± 17.29 U/mL	135	NR	NR	88.9 ± 6.16 U/mL	UA
Bayr A et al. Turkey 2015, (a) [27]	100 #	NR	NR	1.1 ± 0.2 U/mL	64	NR	NR	1.2 ± 0.9 U/mL	STEMI
Bayr A et al. Turkey 2015, (b) [27]	100 #	NR	NR	1.1 ± 0.2 U/mL	31	NR	NR	1.1 ± 0.4 U/mL	NSTEMI
Bayr A et al. Turkey 2015, (c) [27]	100 #	NR	NR	1.1 ± 0.2 U/mL	5	NR	NR	1.0 ± 0.4 U/mL	UA
Mehta MD et al. India, 2015 [28]	45 #	NR	NR	45.11 ± 8.53 U/mL	45	NR	NR	121.09 ± 41.15 U/mL	ACS
Akgöl E et al. Turkey, 2016 [29]	61 #	59	47/14	0.534 ± 0.116 ABSU	63	61	49/14	0.644 ± 0.168 ABSU	ACS
Mishra B et al. Nepal, 2018 (a) [30]	50 #	NR	NR	0.410 ± 0.081 ABSU	14	NR	NR	0.843 ± 0.146 ABSU	STEMI
Mishra B et al. Nepal, 2018 (b) [30]	50 #	NR	NR	0.410 ± 0.081 ABSU	8	NR	NR	0.925 ± 0.094 ABSU	NSTEMI
Mishra B et al. Nepal, 2018 (c) [30]	50 #	NR	NR	0.410 ± 0.081 ABSU	28	NR	NR	0.783 ± 0.221 ABSU	UA
Demir MT et al. Turkey, 2018 (a) [31]	20 #	27	14/6	9.9 ± 1.8 IU/mL	20	59	17/3	18.6 ± 12.2 IU/mL	STEMI
Demir MT et al. Turkey, 2018 (b) [31]	20 #	27	14/6	9.9 ± 1.8 IU/mL	20	64	15/5	16.5 ± 5.2 IU/mL	NSTEMI
Demir MT et al. Turkey, 2018 (c) [31]	20 #	27	14/6	9.9 ± 1.8 IU/mL	20	53	17/3	21.2 ± 16.2 IU/mL	UA
Gholikhani-Darbroud R et al. Iran, 2018 [32]	52 #	60	26/26	0.394 ± 0.227 ABSU	52	63	26/26	0.828 ± 0.328 ABSU	NSTEMI
Mojibi N et al. Iran, 2018 (a) [33]	25 #	62	13/12	0.535 ± 0.037 ABSU	25	58	17/8	0.575 ± 0.086 ABSU	STEMI
Mojibi N et al. Iran, 2018 (b) [33]	25 #	62	13/12	0.535 ± 0.037 ABSU	25	64	10/15	0.609 ± 0.119 ABSU	NSTEMI
Mojibi N et al. Iran, 2018 (c) [33]	25 #	62	13/12	0.535 ± 0.037 ABSU	25	63	14/11	0.834 ± 0.111 ABSU	UA
Choudhury TZ et al. Bangladesh, 2019 [34]	70 #	46	NR	1.38 ± 0.06 U/mL	70	54	NR	2.11 ± 0.08 U/mL	ACS
Yang F et al. China, 2019 (a) [35]	60 #	60	32/28	70.75 ± 3.14 U/mL	64	65	44/20	76.56 ± 3.15 U/mL	STEMI
Yang F et al. China, 2019 (b) [35]	60 #	60	32/28	70.75 ± 3.14 U/mL	56	65	37/19	74.6 ± 3.17 U/mL	NSTEMI
Yang F et al. China, 2019 (c) [35]	60 #	60	32/28	70.75 ± 3.14 U/mL	60	64	39/21	72.86 ± 3.78 U/mL	UA
Aladağ N et al. Turkey, 2021 (a) [36]	55 #	56	29/26	900 ± 100 U/L	50	58	39/11	2400 ± 100 U/L	STEMI
Aladağ N et al. Turkey, 2021 (b) [36]	55 #	56	29/26	900 ± 100 U/L	55	60	37/18	1800 ± 300 U/L	NSTEMI
Özbiçer S et al.Turkey, 2021 [37]	61 ^	61	35/26	0.28 ± 0.04 ABSU	162	58	98/64	0.47 ± 0.10 ABSU	NSTEMI

Legend: NR, not reported; ABSU, absorbance units; IU, international units; U, units; ACS, acute coronary syndrome; AMI, acute myocardial infarction; NSTEMI, non-ST-elevation myocardial infarction; STEMI, ST-elevation myocardial infarction; *, mean or median; #, healthy controls; ^, healthy controls with atypical chest pain.

## Data Availability

The data that support the findings of this systematic review and meta-analysis are available from the corresponding author, A.Z., upon reasonable request.

## Supplementary Materials

The following are available online at https://www.mdpi.com/article/10.3390/jcm11144205/s1, Table S1: PRISMA 2020 abstract checklist; Table S2: PRISMA 2020 manuscript checklist; Table S3: The Joanna Briggs Institute critical appraisal checklist.
