# Supplementary material for: Serum Concentrations of Ischaemia-Modified Albumin in Acute Coronary Syndrome: A Systematic Review and Meta-Analysis
Source: J Clin Med. 2022 Jul 20;11(14):4205. doi: 10.3390/jcm11144205 (PMC9324639; doi:10.3390/jcm11144205)
Supplement: Supplementary file 1 [file jcm-11-04205-s001.zip › Supplementary Table S3.pdf]

Supplementary Table S3. The Joanna Briggs Institute critical appraisal checklist.

Study	Were the criteria for inclusion clearly defined?	Were the subjects and the setting described in detail?	Was the exposure measured in a valid and reliable way?	Were objective, standard criteria used for measurement of the condition?	Were confounding factors identified?	Were strategies to deal with confounding factors stated?	Were the outcomes measured in a valid and reliable way?	Was appropriate statistical analysis used?	Risk of bias
Abadie [21]	Yes	Yes	Yes	Yes	No	No	Yes	No	Low
Aparci [22]	Yes	Yes	Yes	Yes	No	No	Yes	No	Low
Ju [23]	Yes	Yes	Yes	Yes	No	No	Yes	No	Low
Dawie [24]	Yes	Yes	Yes	Yes	No	No	Yes	No	Low
Ertekin [25]	Yes	Yes	Yes	Yes	No	No	Yes	No	Low
Patil [26]	Yes	Yes	Yes	Yes	No	No	Yes	No	Low
Gurumurthy [11]	Yes	Yes	Yes	Yes	No	No	Yes	No	Low
Bayr [27]	Yes	Yes	Yes	Yes	No	No	Yes	No	Low
Mehta [28]	Yes	Yes	Yes	Yes	No	No	Yes	No	Low
Akgol [29]	Yes	Yes	Yes	Yes	No	No	Yes	No	Low
Mishra [30]	Yes	Yes	Yes	Yes	No	No	Yes	No	Low
Demir [31]	Yes	Yes	Yes	Yes	No	No	Yes	No	Low
Gholikhani-Darbroud [32]	Yes	Yes	Yes	Yes	No	No	Yes	No	Low
Mojibi [33]	Yes	Yes	Yes	Yes	No	No	Yes	No	Low
Choudhury [34]	Yes	Yes	Yes	Yes	No	No	Yes	No	Low
Yang [35]	Yes	Yes	Yes	Yes	Yes	Yes	Yes	Yes	Low
Aladag [36]	Yes	Yes	Yes	Yes	No	No	Yes	No	Low
Ozbicer [37]	Yes	Yes	Yes	Yes	No	No	Yes	No	Low
